# Alcohol Induced Brain and Liver Damage: Advantages of a Porcine Alcohol Use Disorder Model

**DOI:** 10.3389/fphys.2020.592950

**Published:** 2021-01-07

**Authors:** Soo K. Shin, Erin E. Kaiser, Franklin D. West

**Affiliations:** ^1^Regenerative Bioscience Center, University of Georgia, Athens, GA, United States; ^2^Interdisciplinary Toxicology Program, University of Georgia, Athens, GA, United States; ^3^Department of Animal and Dairy Science, College of Agricultural and Environmental Sciences, University of Georgia, Athens, GA, United States; ^4^Neuroscience Program, Biomedical and Health Sciences Institute, University of Georgia, Athens, GA, United States

**Keywords:** porcine (pig) model, alcohol, alcohol use disorder, voluntary alcohol intake/consumption, brain, liver, alcohol injury, alcohol metabolism

## Abstract

Alcohol is one of the most commonly abused intoxicants with 1 in 6 adults at risk for alcohol use disorder (AUD) in the United States. As such, animal models have been extensively investigated with rodent AUD models being the most widely studied. However, inherent anatomical and physiological differences between rodents and humans pose a number of limitations in studying the complex nature of human AUD. For example, rodents differ from humans in that rodents metabolize alcohol rapidly and do not innately demonstrate voluntary alcohol consumption. Comparatively, pigs exhibit similar patterns observed in human AUD including voluntary alcohol consumption and intoxication behaviors, which are instrumental in establishing a more representative AUD model that could in turn delineate the risk factors involved in the development of this disorder. Pigs and humans also share anatomical similarities in the two major target organs of alcohol- the brain and liver. Pigs possess gyrencephalic brains with comparable cerebral white matter volumes to humans, thus enabling more representative evaluations of susceptibility and neural tissue damage in response to AUD. Furthermore, similarities in the liver result in a comparable rate of alcohol elimination as humans, thus enabling a more accurate extrapolation of dosage and intoxication level to humans. A porcine model of AUD possesses great translational potential that can significantly advance our current understanding of the complex development and continuance of AUD in humans.

## Introduction

Although alcohol is one of the most preventable causes of death in the United States, it causes 88,000 deaths each year and claims the life of 1 in 10 working age adults (Mokdad et al., 2004; Gonzales et al., 2014). Fatalities, health care expenditures, and costs associated with the loss of workplace productivity due to alcohol consumption yields an economic burden of $250 billion annually in America. Approximately 15.1 million adults (≥ 18 years of age) and 623,000 adolescents (12–17 years of age) suffer from alcohol use disorder (AUD) in United States with numbers on the rise (National Institute on Alcohol Abuse and Alcoholism, 2020). Binge or excessive drinking is the most common, costly, and deadly pattern of alcohol use (Gonzales et al., 2014). The National Institute of Alcohol Abuse and Alcoholism defines binge drinking (BD) as 4 or 5 drinks per occasion for women and men, respectively, or alcohol consumption that results in a blood alcohol concentration (BAC) of 0.08% (American Addiction Centers, 2019). BD does not directly equate to AUD, but heavy drinkers (defined as users that BD ≥ 5 times a month) are significantly more likely to develop AUD (American Addiction Centers, 2019). The at risk population for AUD is surprisingly large as 1 in 6 adults BD 4 times in a month (Kanny et al., 2018).

Due to the high prevalence of AUD, rodent models have been developed and extensively studied since the 1940s (Crabbe, 2016). While rodent models have been instrumental in understanding the molecular and genetic implications of AUD, they have a number of limitations. Rodents generally have a low preference for alcohol and will not voluntarily drink to intoxication. Alcohol must often be administered by oral gavage, vaporization, infusion, alcohol diet, or intravenous, intragastric, or intraperitoneal injections to study the physiological effects (Crabbe et al., 2011). However, AUD is a mental disorder, and thus requires voluntary consumption to better understand the progression of the disorder. A defining characteristic of AUD is continued alcohol usage despite negative consequences, which cannot be studied in forced consumption paradigms (American Psychiatric Association, 2013). To overcome this challenge, researchers have implemented several tactics to encourage voluntary consumption in rodents, including selective breeding and food and water deprivation (Crabbe et al., 2011). However, these methods introduce many confounders as it primes animals for alcohol abuse and disregards the heterogeneous nature of the human population, which makes studying risk factors involved in excessive alcohol consumption difficult (Grant and Bennett, 2003). These studies are useful in exploring the physical implications of alcohol abuse, but not in understanding the development and progression of the disorder.

Comparatively, the pig is an attractive alternative to model human alcoholism due to behavioral, anatomical, and physiological similarities to humans. In early pig studies, animals were given free access to water and alcohol in aqueous solution (Dexter et al., 1976; Tumbelson et al., 1979; Tumbleson et al., 1981a, b). Pigs underwent two bottle choice testing (2BC) in a chronic *ad libitum* exposure. During this period, pigs displayed intoxication behaviors characterized by ataxia, extreme passivity, state of consciousness alternation, and occasional vomiting. Intoxication was also confirmed by measuring ethanol levels in the blood plasma. Despite negative consequences, these animals continued to drink within the session in which the negative consequences were observed and during later drinking sessions. After animals were no longer allowed to have access to alcohol, withdrawal symptoms such as static and volitional tremor, dilated pupils, and muscle fasciculation were observed. These studies showed that pigs displayed continued voluntary alcohol consumption despite experiencing the negative effects of alcohol intoxication. These studies demonstrated that pigs voluntarily consumed alcohol to intoxication and showed withdrawal symptoms analogous to humans, which contrasts with non-augmented rodent models. In a recent study from our group, the effects of voluntary alcohol consumption on motor function were examined in a 2BC (Shin et al., 2020). In this porcine model of binge drinking, high blood alcohol levels (BAL) were achieved by voluntary oral consumption and animals exhibited perturbation in gait parameters that were also affected in humans during alcohol intoxication. Pigs with high BAL of ≥ 150 mg/dl displayed severe to complete loss of coordination. Despite these negative consequences of alcohol intoxication, pigs continued to consume alcohol during the same and later drinking sessions. More studies are needed to examine the true addictive behavior in porcine models, but these models showed continued alcohol consumption despite negative consequences (i.e., ataxia, motor deficits, state of consciousness alteration, and vomiting). In addition, the pig has comparable brain anatomy including gyrification, structures, gray/white matter composition, and vasculature, which likely yields similar alcohol induced injury patterns as different tissues of the brain display differential sensitivity to alcohol. Pigs also have similar liver anatomy and physiology as well as body size that results in a comparable rate of alcohol metabolism. On the contrary, differential liver physiology and relatively small size of rodents give rise to a rapid rate of alcohol metabolism relative to humans.

**FIGURE 1:**
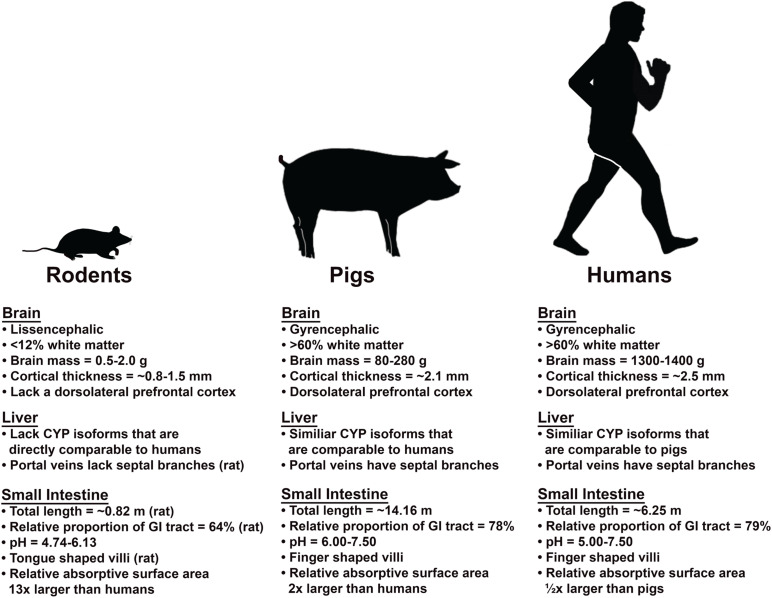
Comparison of the brain, liver, and small intestine of rodent and pig models relative to humans. Pigs and humans share many similarities in brain, liver and small intestine that results in similar absorption, metabolism/excretion, and intoxication processes.

In this review, we examine the mechanistic effect of alcohol on key structures and functional systems in the brain and liver in humans, rodents, and pigs. Furthermore, we discuss the limitations of current rodent models in studying AUD mechanisms in the brain and liver and examine the porcine model as a potentially more representative animal model due to intrinsic behavioral, anatomical, and physiological attributes (Figure 1).

### Search Strategy and Selection Criteria

The articles in this review were retrieved using the following electronic databases: PubMed, MEDLINE, Google Scholar, and Web of Science. The search was limited to articles published between May 1, 1976 and September 13, 2019. Search terms were identified in the title, abstract, and key words using the following search terms: alcoholism, alcohol use disorder, alcohol/ethanol, pig/porcine/swine, rodent, rat, mouse, animal model, alcohol induced damage, oxidative stress, inflammation, immune response, mechanism, prefrontal cortex, limbic system, hippocampus, amygdala, alcohol dependence, chronic/acute exposure, and alcohol consumption.

## Alcohol Alters the Morphology and Physiology of the Brain

### Alcohol Induced Brain Damage Is Multi-Mechanistic With a Synergistic Combination of Insults

Neuropathology of alcohol induced damage is not exclusive to any one mechanism but is instead the result of a synergistic combination of insults that lead to a global disruption in normal brain function. When humans engage in drinking, the brain is subjected to heavy alcohol exposure due to a rich blood supply (Paton, 2005). Damage to the brain is mainly mediated by increased oxidative stress and proinflammatory cytokines, thus creating a neurotoxic environment and consequently, neurodegeneration. Alcohol induced oxidative stress is initiated when alcohol is oxidized to acetaldehyde either by alcohol dehydrogenase or Cytochrome P450-2E1 (CYP2E1) (Cederbaum, 2012). This metabolite is then oxidized to acetate by aldehyde dehydrogenase. CYP2E1 mediated alcohol metabolism results in acetaldehyde and H_2_O_2_, which react with copper and iron to produce reactive oxygen species (ROS) (Haorah et al., 2008). As a result of accumulation and reaction with other compounds by H_2_O_2_ and superoxide anion, reactive metabolites are formed. These metabolites cause lipid peroxidation of cell membranes (Louvet and Mathurin, 2015). Alcohol induced oxidative stress in the brain is exacerbated by downregulation of antioxidant enzyme activity of catalase and superoxide dismutase as well as increases in nitric oxide and NADPH oxidase production (Teixeira et al., 2014; Fernandes et al., 2018). In human and rat brains, alcohol exposure resulted in increased CYP2E1 activity and subsequent blood-brain barrier (BBB) breakdown (Haorah et al., 2005). Metabolism of alcohol by CYP2E1 in the brain microvascular endothelial cells activated myosin light chain kinase, which in turn phosphorylated myosin light chain and tight junction proteins, thus altering the integrity of cytoskeleton and tight junctions. Decreased BBB integrity then elicited a neuroinflammatory response by allowing monocyte migration across the BBB.

Detailed investigation of molecular mechanisms in the rat brain revealed that alcohol activates a complex inflammatory signaling cascade that leads to apoptosis in neurons and glia (Crews and Nixon, 2009; Liu et al., 2017). Alcohol exposure resulted in increased DNA binding of the inflammatory factor nuclear factor κB (NF-κB) and decreased binding of the pro-survival factor cAMP responsive element-binding protein (CREB) (Crews and Nixon, 2009). NF-κB regulates inflammatory and immune responses and activation of NF-κB transcription elicited the production of proinflammatory cytokines including TNF-α, IL-1β, IL-6, and IL-12 (Silswal et al., 2005; Crews and Nixon, 2009; Liu et al., 2017). On the contrary, CREB is crucial for neuronal survival and regulates transcription of survival factors. This neuroprotective protein shields cells from excitotoxicity and inhibits apoptosis signaling; disruption of its function in the brain leads to neurodegeneration (Lonze and Ginty, 2002). CREB activation was decreased during alcohol intoxication from binge and chronic alcohol consumption. Decreased levels of activated CREB highly correlated with brain regions that displayed signs of neurodegeneration (Bison and Crews, 2003). Figures 4 and 8 in the works of Haorah et al. (2008) and Crews and Nixon (2009), respectively, demonstrate a comprehensive illustration of the mechanisms of alcohol induced brain damage discussed in this section.

Alcohol is detrimental to the brain not only because of its neurodegenerative effects, but also because it inhibits neurogenesis (Crews and Nixon, 2003; Herrera et al., 2003; He et al., 2005). Adult neurogenesis occurs in two brain regions, the hippocampus and olfactory bulb (Crews and Nixon, 2003). Interestingly, quantification of proliferating cells with bromodeoxyuridine revealed that alcohol selectively impeded neurogenesis in the hippocampus, a region of the brain that is particularly sensitive to alcohol insult, by 50–70% (Nixon and Crews, 2002; Herrera et al., 2003). Chronic alcohol exposure also impaired neuronal maturation in the adult rat brain as indicated by the decreased dendritic outgrowth of newborn neurons (He et al., 2005). The characterization of nascent doublecortin positive neurons showed decreased numbers of dendritic nodes and endings, as well as decreased total apical dendrite length. Oxidative stress is likely the cause of neurogenesis inhibition since the antioxidant ebselen was able to provide strong protection against neurogenesis inhibition (Herrera et al., 2003). Most of the mechanistic studies of alcohol induced injuries have been conducted in traditional rodent models but using an animal model with a more comparable brain structure would offer a more representative recapitulation of alcohol injury patterns observed in the human brain.

### Alcohol Causes Differential Damage to White and Gray Matter

Magnetic resonance imaging (MRI) is the most common modality used to study alcohol induced brain injury and has revealed that alcohol consumption leads to neuronal loss and a subsequent reduction in white matter (WM) and gray matter (GM) (Agartz et al., 2003; Cardenas et al., 2007; McQueeny et al., 2009). Neuropsychological performance correlated with a reduction in GM and WM in different regions of the brain, signifying a significant GM and WM tissue loss that can have long-term effects due to alcohol consumption (Hommer, 2003). There is a regional selectivity of alcohol induced damage for GM and WM. Both GM and WM losses were observed in the anterior superior vermis, while the cerebellar hemisphere only displayed a GM reduction (Sullivan et al., 2000). Motor deficits measured by gait and balance tests significantly correlated only with WM loss in the anterior-superior vermis. Gender differences were also observed with respect to alcohol induced WM and GM volume changes (Hommer et al., 2001; Hommer, 2003). Alcoholic women showed a significant reduction in WM and GM volumes relative to controls. Although alcoholic men also showed a significant reduction in WM and GM, the significance for men was a magnitude smaller, thus suggesting women are more vulnerable to alcohol induced damage.

Another volumetric measure of changes in GM and WM is cortical thickness. The pattern of abusive alcohol consumption strongly correlated with neurotoxicity that interfered with normal neuromaturation in the frontal cortex, thus resulting in altered microarchitectural pruning (Mashhoon et al., 2014). Chronic alcoholics, adolescents that practiced intermittent alcohol consumption, and abstinent alcoholics all had thinner cortices than healthy age-matched control groups (Oishi et al., 1999; Fortier et al., 2011; Squeglia et al., 2012). Adolescent alcohol users had decreased cortical thickness and reduced WM integrity compared to non-users, although the groups did not differ at baseline assessment, when users had no prior alcohol experience and had similar premorbid characteristics (Luciana et al., 2013). Changes in cortical thickness due to alcohol consumption also showed gender differences. Adolescent females with recent BD had ∼8% thicker cortices in the left frontal region than female non-drinkers, while adolescent males with BD had ∼7% thinner cortices, thus suggesting the presence of gender specific GM effects (Squeglia et al., 2012).

A major question regarding these studies remains: is a reduction in GM volume, and thus the cortical thickness, a risk factor or a consequence of alcohol consumption? A recent study showed that individuals with a family history of AUD had thinner cortices in the frontal and parietal lobes as opposed to individuals with no history (Henderson et al., 2018). Subjects from both groups had no or limited access to alcohol, eliminating the effects of alcohol as a confounder. A large neuroimaging study with 2,423 individuals also found a smaller right dorsolateral prefrontal cortex and insula GM volumes were associated with increased alcohol use, thus providing further support that a reduction in cortical thickness may be an AUD risk factor (Baranger et al., 2019). Family based and prospective longitudinal data suggested this association is of genetic origin and that smaller GM volumes could serve as a prognostic biomarker for AUD. The authors argued that a reduction in the GM volume is not caused by alcohol consumption, but instead promoted alcohol use, which in turn further potentiated GM loss. The relationship between alcohol consumption and genetic predisposition is bilateral as one influences the other in the development of AUD.

The directionality of GM alteration showed contrasting results (Doallo et al., 2014; Sousa et al., 2017). Where most of the previously noted studies showed decreased GM volume was associated with alcohol consumption, the opposite has also been found to be true. Structural MRI analysis by voxel-based morphometry on human subjects with persistent (≥ 3 years) BD patterns showed larger GM volumes in the left mid-dorsal cortex compared to controls. This study proposed a relationship between thicker cortices and less neurodevelopment, as indicated by a positive correlation between cortical thickness and error scores in a self-ordering pointing test, a test designed to assess planning and self-monitoring aspects of the working memory (Doallo et al., 2014). Another study observed a similar increase in cortical thickness in the left middle frontal gyrus of adolescents practicing BD for ≥ 10 months (Sousa et al., 2017). It is interesting to note that both of these studies analyzed adolescent subjects and differences were seen in the prefrontal cortex, which is the last area of the brain to fully develop (Doallo et al., 2014). Adolescence is a critical time in brain development and is a window of high alcohol sensitivity.

Some of the factors that may be involved with WM volume reduction include axonal atrophy, cellular membrane breakdown, and demyelination (de la Monte, 1988; Lewohl et al., 2000). Conventional MRI sequences may not sufficiently detect these changes, possibly contributing to the general perception of less WM damage relative to GM. Recent MRI technological developments are being increasingly implemented in clinical settings, such as diffusion tensor imagining (DTI), which enables the assessment of more sensitive WM parameters in order to survey more subtle changes (Pfefferbaum and Sullivan, 2005). For example, DTI-derived fractional anisotropy (FA), a parameter that measures the orientation and coherence of WM, was decreased in alcoholic women compared to controls despite the lack of differences in structural MRI sequences between groups (Pfefferbaum and Sullivan, 2002). Adolescents that practice BD and alcoholic men also showed a decrease in WM FA (Pfefferbaum and Sullivan, 2005; McQueeny et al., 2009). Additionally, DTI-derived radial diffusivity (RD) showed a significant increase in heavy drinkers relative to light drinkers for middle aged men (McEvoy et al., 2018). This increase in RD suggests decreased myelination in heavy drinkers (McEvoy et al., 2018).

WM is potentially more sensitive to alcohol induced damage due to lower intrinsic antioxidant properties and the differential response of its vasculature to alcohol. Oligodendrocytes are at high risk for oxidative damage as they have limited defense and repair mechanisms (Sasaki and Senda, 1999; Salminen and Paul, 2014). Rat oligodendrocyte precursor cells (OPC) subjected to oxidative stress *in vivo* showed disrupted WM repair caused by disturbed OPC renewal mechanisms. In addition, the WM vasculature is more affected by alcohol than GM vasculature, showing a stronger correlation between alcohol induced increase in cerebral blood flow (CBF) and volume than GM in social drinkers (Gundersen et al., 2013). This increased CBF leads to increased WM alcohol exposure and damage. A related study found that individuals with a low response to alcohol intoxication displayed lower CBF changes than high response individuals, despite reaching the same blood alcohol level, thus indicating CBF plays a crucial role in alcohol intoxication (Tolentino et al., 2011). Although conventional anatomical sequences may fail to reflect higher sensitivity of alcohol induced damage in WM, there is significant evidence to indicate heightened vulnerability. Different structure of GM and WM result in differential vulnerability to alcohol induced damages, thus demonstrating the need for an animal model that has a similar composition of WM and GM of the brain as humans.

### Prefrontal Lobe and Corticolimbic System

The prefrontal lobe work in conjunction with the corticolimbic system as an important regulator of executive function, emotion, memory, behavior, and reward and stress systems that regulate addictive behaviors (Chanraud et al., 2007; Bergstrom and Pinard, 2017). The development and continuance of AUD is associated with a decreased volume in the reward structures of the prefrontal lobe and corticolimbic system including the hippocampus and amygdala (Nixon and McClain, 2010; Leisman et al., 2012). Sustained heavy drinking in college students has been associated with accelerated GM volume loss in the prefrontal cortex and hippocampus (Meda et al., 2018). Chanraud et al. (2007) reported that the brains of recovering alcoholic men had up to a 20% reduction in GM, primarily in the dorsolateral frontal cortex. Comparatively, WM volume reduction was more widespread, but resulted in a lesser total volume loss of 10%. Additionally, abnormally altered volumes of these structures were associated with decreased memory scores, increased alcohol cravings, and vulnerability to relapse (Makris et al., 2008; Wrase et al., 2008). The following sections discuss the significance of specific brain structures as they pertain to AUD and their heightened vulnerability to alcohol induced injury.

#### Prefrontal Lobe

Alcohol exposure results in reduced prefrontal lobe volume and cognitive deficits. With significant GM and neuronal loss, it is one of the most affected regions of the alcoholic brain (Fein et al., 2002; Sullivan and Pfefferbaum, 2005; Doallo et al., 2014). There was a negative correlation between prefrontal cortex GM volumes and alcohol consumption frequency, average number of drinks, maximum number of drinks per episode, age of onset, and lifetime duration for AUD (Fein et al., 2002; De Bellis et al., 2005). More specifically, the prefrontal dorsolateral cortex volume was highly associated with alcohol dependence and the amount of consumption as well as a decrease in both density and size of glia (Miguel-Hidalgo et al., 2002; Chanraud et al., 2007; Makris et al., 2008; Baranger et al., 2019). Negative correlations in the volume of both global and regional GM in the bilateral frontal gyri is associated with lifetime alcohol intake in social drinkers as well (Taki et al., 2006). The frontal lobe is known to control executive functions such as motivation, planning, and inhibition of impulsive responses. Damage to the frontal lobe results in the loss of executive function and behavioral problems that enhance addiction (Crews and Boettiger, 2009). For example, injury to the human and rat orbital frontal cortex has been shown to increase the proclivity to choose immediate rewards over larger, delayed rewards (Rudebeck et al., 2006). A vicious cycle of frontal lobe damage by alcohol encourages behaviors associated with addiction, and subsequent increase in consumption further exacerbates frontal lobe injury.

In the frontal lobe WM, ^1^H magnetic resonance spectroscopy demonstrated that recently detoxified alcoholics had alterations in N-acetylaspartate (NAA) and myo-inositol (INO) levels. There was a 14.7% reduction of NAA in the frontal WM while those of parietal WM were similar to healthy controls, thus suggesting selective damage to the frontal lobe (Schweinsburg et al., 2001). NAA serves as a robust biomarker of neural density and health as it is found exclusively in neurons and is highly sensitive to injury (Wellard et al., 2004). INO is an intracellular second messenger that acts as an osmolyte and a marker of astrocyte density (Wellard et al., 2004). An 11.8% increase in INO was observed in recently detoxified alcoholics, possibly indicating astrocyte proliferation and an osmotic response to cell shrinkage due to degeneration of neurons (Schweinsburg et al., 2001). In addition, whole brain arterial blood imaging and positron emission tomography (PET) demonstrated that acute exposure causes increased CBF, particularly in the frontal brain region (Tolentino et al., 2011). This could explain why the frontal lobe sustains greater brain damage, as alcohol rapidly diffuses from vessels to tissues.

#### Hippocampus

The hippocampus plays a major role in learning, memory, and spatial navigation, all of which are negatively affected by alcohol intoxication (Anand and Dhikav, 2012). Alcohol disrupts the ability of the hippocampus to form explicit memory, a type of long term memory (White, 2003). Inhibition of this type of memory can lead to blackouts if large amounts of alcohol are consumed. An alcoholic blackout is defined by the inability to recall any events during intoxication without losing consciousness. Although the exact mechanism is unknown, alcohol is known to interfere with encoding short term memory to long term memory by inhibiting the activity of CA1 pyramidal neurons of the hippocampus (Lee et al., 2009). In a recent MRI study, examination of human patients showed that the hippocampal volumes were significantly smaller in patients with AUD relative to healthy controls (De Bellis et al., 2000; Hommer et al., 2001; Meda et al., 2018). Additionally, the total hippocampal volume was positively correlated with the age of onset of AUD and negatively with the duration of AUD and memory scores (De Bellis et al., 2000; Meda et al., 2018). In rodent studies, only 1 day of BD induced a two to eightfold upregulation of reactive gliosis and a two to ninefold increase in neurodegeneration in the rat hippocampus (Hayes et al., 2013). The hippocampus is unique in that it continues neurogenesis in adulthood, but alcohol hinders this process (Nixon and Crews, 2002; He et al., 2005). Months of abstinence can restore neurogenesis and the formation of new neurons in the hippocampal dentate gyrus (Crews and Nixon, 2009).

#### Amygdala

The amygdala plays an integrative role in emotional responses, sexual behavior, and detecting environmental danger. It is involved in many emotional disorders in humans including anxiety, social phobia, schizophrenia, and bipolar disorder (Schumann et al., 2011). In humans, the amygdala volume has been associated with the cravings and relapse of alcohol consumption (Nixon and McClain, 2010). A significant decrease in amygdala volume was observed in alcohol dependent subjects and was associated with increased alcohol cravings and prospective relapse 6 months after abstinence (Fein et al., 2006; Wrase et al., 2008; Schumann et al., 2011). Furthermore, there was no difference in the amygdala volume for current vs. past alcoholics, although individuals with lifetime AUD possessed smaller volumes than healthy counterparts (Dager et al., 2015). This suggests long-term damage that shows no recovery after abstinence or that small amygdala volume could serve as a risk factor. Interestingly, there is mounting evidence that infers a smaller amygdala as a risk factor for AUD, instead of a neurotoxic effect of alcohol. Many high-risk subjects (i.e., individuals with a family history of AUD) have markedly reduced amygdala volume compared to healthy controls—even if the individual was considered alcohol-naïve. This suggests that the amygdala volume is genetically correlated with the likelihood of AUD, predisposing one to severe alcohol consumption (Fein et al., 2006; Benegal et al., 2007; Dager et al., 2015). More research is needed to understand the relationship between amygdala volume and alcohol consumption.

### Mesolimbic System

The mesolimbic system is a collection of dopaminergic neurons that regulate motivated behaviors, cognitive processes, and response to reward. Dopamine (DA) and serotonin (5-HT), a key modulator of DA neurons in all 3 major dopaminergic pathways, have long been associated with drug abuse (Alex and Pehek, 2007). DA and 5-HT are the main neurotransmitters (NT) that act as a driving force for the rewarding effects of alcohol. While drinking initially boosts the levels of these NTs, repeated use reduces sensitivity for NT release, thus ultimately resulting in lower levels (Wise and Rompre, 1989; Volkow et al., 1996; Belmer et al., 2016). Significantly higher striatal DA and 5-HT transporters were observed during acute withdrawal in alcoholics, further decreasing the availability of these NTs (Cosgrove et al., 2009). However, there are some differences in how these NTs interact with alcohol.

#### Serotonin Systems

The serotonin system is crucial in regulating mood, particularly stress and anxiety, which significantly affect alcohol consumption (Belmer et al., 2016). P rats, a rat strain selectively bred for preference of alcohol over water, have fewer serotonergic neurons than normal rats, and therefore have reduced baseline levels of 5-HT and its metabolites. Low serotonergic activity has been implicated in anxiety and depression, possibly contributing to the innately high levels of anxiety and alcohol consumption observed in P rats (De Vry, 1995; Albert et al., 2014). Human alcoholics and animal models displayed reduced alcohol consumption when treated with selective 5-HT reuptake inhibitors, with humans reporting less desire and pleasurable effects of alcohol (LeMarquand et al., 1994; Pettinati, 1996; Lovinger, 1997). This supports the hypothesis that P rats consume high volumes of alcohol to normalize brain 5-HT levels, since acute alcohol exposure can elevate 5-HT levels (Lovinger, 1997). Notably, a clinical study demonstrated that humans with alcohol dependence display an upregulation of 5-HT_1B_ receptors in the ventral striatum (Hu et al., 2010). The implication of 5-HT_1B_ receptors effecting alcohol dependency has been observed in rodent models as well. In fact, a strain of 5-HT_1B_ receptor knockout mice exhibited less intoxication in response to alcohol compared with the normal mice, suggesting the role of 5-HT_1B_ receptor in producing some of alcohol’s intoxication symptoms (Crabbe et al., 1996).

#### Dopamine Systems

The DA system has been linked with reinforcing properties of alcohol abuse and the administration of DA receptor antagonists in the nucleus accumbens attenuates alcohol self-administration in rats (Wise and Rompre, 1989; Rassnick et al., 1992). Repeated alcohol use resulted in reduced DA production and receptors in human alcoholics with D2 receptors further reinforcing the effects of alcohol (Stefanini et al., 1992; Volkow et al., 1996; Nowak et al., 2000). In humans, PET scans showed that individuals with AUD had a 20% reduction in striatal D2 receptor availability and affinity compared to controls, demonstrating the relationship between reduced density and sensitivity of the receptors in the limbic system with preference for alcohol (Hietala et al., 1994; Volkow et al., 1996). In parallel, P rats have fewer D2 receptors in the limbic system relative to wildtype rats (Stefanini et al., 1992; McBride et al., 1993). Thanos et al. (2001) found that adenovirus vector mediated overexpression of D2 receptors reduced alcohol self-administration by 64% and preference by 43% in P rats. This suggests that D2 receptor density plays a role in mediating alcohol consumption and preference in rats. Lower levels of DA release in individuals with AUD could be from desensitization to the effects of alcohol over a long period of abuse. These results indicate that lower levels of DA and its receptors are AUD risk factors.

## Discussion: A Translational Porcine Model Provides Potential Advantages in Recapitulating Human AUD

Since wild type rodents have a low preference for alcohol, many methods have been implemented to model human alcohol consumption including selective breeding, induction of dependence, and water and food deprivation (Crabbe et al., 2011). However, these rodent models do not fully represent human behavior and consequently, cannot recapitulate the complexity of AUD development and the progression of behavioral, psychological, and physiological factors. However, pigs drink alcohol to intoxication without manipulation, thus enabling improved AUD studies (Dexter et al., 1976; Tumbelson et al., 1979). In addition, porcine models are often used in medical research due to their anatomical, physiological, and biochemical similarities to humans (Bode et al., 2010; Nykonenko et al., 2017). Porcine models also have practical advantages over other large animal models such as non-human primates as they possess significantly less financial and regulatory burden. Furthermore, non-human primates do not voluntarily consume alcohol to intoxication and require induction (Macenski and Meisch, 1992). The advantages of a porcine model in studying the mechanism of alcohol induced brain and liver damage will be discussed in detail.

### Similar Size and Anatomy of Pigs Result in Comparable Rate of Alcohol Metabolism

Mammals with smaller body masses generally metabolize alcohol much faster than larger animals due to higher basal metabolic rates. For example, mice eliminate alcohol 5 times faster than humans (Addolorato et al., 1997; Cederbaum, 2012). Therefore, the volume of alcohol used in rodent models to reach intoxication is often several fold higher than in humans (7–13 vs. 0.7–1 g/kg, respectively) (Crabbe et al., 2011; Hayes et al., 2013; Waszkiewicz et al., 2018). Moreover, rodents have shorter life cycles, so the duration of alcohol exposure would correspond to a longer period of alcohol consumption in humans. As a result, extrapolation of findings in rodent models to humans must be done with significant consideration given species-specific differences. However, pigs have a similar metabolism rate relative to humans in part due to analogous body masses. Miniature pig breeds commonly used for biomedical research achieve maximum weights ranging from 70 to 140 kgs- a size more analogous to humans (Reiland, 1978). In addition to similar body mass, endocrine, cardiovascular, and other internal organ systems are also more comparable to humans. Of particular interest is the gastrointestinal (GI) tract, where approximately 20% of alcohol is absorbed in the stomach and 80% is absorbed in the small intestine (SI). The anatomy, morphology, and physiology of the pig SI, the primary site of alcohol absorption, is similar to humans in terms of total length, transit time, and pH (Bode et al., 2010). For example, the average total length of the SI and relative proportion of the SI to the entire GI tract length in humans are 6.25 m and 79% as compared to those of pigs at 14.16 m and 78% and 0.82 m and 64% in rats, respectively (Figure 1; Kararli, 1995; Hatton et al., 2015). The estimated absorptive surface area of the SI relative to body weight revealed that pigs have a surface area that is double that of humans, while the SI to body weight ratio for mice is 13 times larger than humans (Hatton et al., 2015; Agoston, 2017). This may in part explain the rapid metabolism of alcohol in mice. Furthermore, the 6–7.5 pH of the pig SI is more analogous to humans at 5–7.5 as opposed to 4.74–6.13 in rodents. Similar pH of the GI is important because it plays a crucial role in the biotransformation of alcohol and enzymatic activities. Since alcohol is absorbed into the body through simple diffusion, the surface area of the GI membrane dictates the rate in which alcohol is absorbed into the body, which plays a role in the metabolism of alcohol. The gross (ratio of intestinal length per kg of body) and microscopic (structure of villi and types of cells that constitute the intestinal epithelium) similarities between the human and pig SI contribute to analogous digestive and absorptive processes (Gonzalez et al., 2015). The villi of humans and pigs SI are finger-shaped while those of the rat is tongue shaped, which result in a differential SI surface area (Hatton et al., 2015). Moreover, the rate of alcohol voluntary consumption, blood elimination, tolerance, dependence, and withdrawal symptoms are similar to humans, thus suggesting the pig could be a highly predictive model to examine the development and mechanisms of AUD (Dexter et al., 1976; Keiding et al., 1979).

### Similar Structure of the Pig and Human Brains Result in Increased Translational Potential of the Porcine AUD Model for Alcohol Induced Brain Damage

Overall, humans and pigs have more comparable brain cytoarchitecture, organization, growth, and development than rodents (Vodicka et al., 2005; Lind et al., 2007). Rodents have small lissencephalic brains, lacking cortical sulci and gyri with a small proportion of WM compared to humans. Humans and pigs both have gyrencephalic brains, which is the result of rapid GM growth unmatched by WM during development, thus leading to cerebral folds and increased cortical surface area to brain mass ratio (Giedd, 2004; Gogtay and Thompson, 2010). The formation of gyri and sulci allows for more compact network connectivity and enhanced neural processing. This increases the cortical surface area to total brain mass ratio and the number of neurons as compared to rodents, which are factors involved in the cortical functional organization and development (Dubois et al., 2008; Gieling et al., 2011). Alterations in gyral and sulcal patterns have been associated with a number of neuropsychiatric disorders (Van Essen et al., 2006; Dubois et al., 2008). Alcohol has been shown to cause cortical thinning in alcoholics and, interestingly, shows differential thinning with greater change in the sulci relative to gyri. Furthermore, greater recovery of sulci cortical thickness was seen after 2 weeks of abstinence in humans, thus suggesting a selective vulnerability of sulci to alcohol exposure and the role it plays in abstinence induced recovery (Wang et al., 2016). Studying the porcine brain, with its similar gyral patterns to humans, would enable a more accurate analysis of the convoluted nature of alcohol induced brain damage. This is not possible in a lissencephalic rodent model that is devoid of gyrification. Larger animals have higher brain mass and neuronal complexity (Roth and Dicke, 2005). The human brain weighs 1,300–1,400 g, as compared to 80–180 g pig brains and 0.5–2 g rodent brains (Hofman, 1985; Lind et al., 2007; Gieling et al., 2011). As alcohol causes neurodegeneration that reduces the mass and volume of the brain, having a comparable brain size would allow for a more direct comparison of alcohol induced brain damage. Moreover, the mass of the brain dictates the amount of CBF at a rate of ∼50 mL for each 100 g of brain tissue per minute. Therefore, CBF controls the amount of alcohol exposure on the brain (Caplan, 2009).

#### Gray and White Matter

**FIGURE 2:**
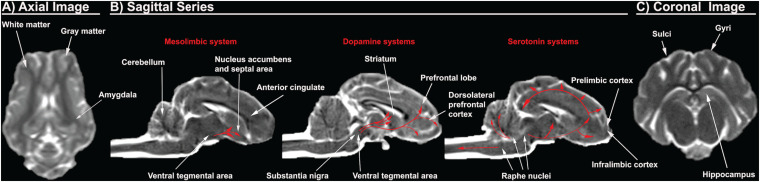
Porcine T2Weighted MRI sequences display critical cerebral structures involved with AUD. An axial slice of the porcine brain displays high white matter composition more comparable to humans than rodents **(A)**. Sagittal sequences display the dorsolateral prefrontal cortex, which is present in pigs and humans, but absent in rodents, as well as anterior cingulate, prelimbic cortex, and infralimbic cortex. The distribution of the dopamine and serotonin systems are traced in red arrows **(B)**. A coronal slice shows gyri and sulci, which are present in pigs and humans, but absent in rodents. The pig and human hippocampus are comparable in terms of structure and orientation **(C)**.

WM and GM are impacted differently by alcohol, with a distinctive regional selectivity for alcohol induced damage (Sullivan et al., 2000; Agartz et al., 2003; Cardenas et al., 2007; Fortier et al., 2011). WM is potentially more susceptible to alcohol induced injury due to decreased levels of defense mechanisms against oxidative stress and increased sensitivity to alcohol induced alterations in the vasculature as indicated by increased CBF and cerebral blood volume due to the vasoactive properties of alcohol (Ravindranath et al., 1989; Sasaki and Senda, 1999; Gundersen et al., 2013; Salminen and Paul, 2014). As such, using an animal model with a similar WM to GM ratio will be critical in understanding alcohol induced neuropathology (Figure 2A). The mature rat brain is comprised of < 12% of WM while the mature human and pig brains have > 60% (Figure 1; Kinder et al., 2019). As alcohol exhibits differential damage on these tissues, having a similar WM to GM ratio would result in a similar pattern of injury on the brain and thus similar manifestation of deficits as a result of the injury. Cortical thickness also decreases as WM and GM degenerate after alcohol exposure (Fortier et al., 2011; Squeglia et al., 2012; Luciana et al., 2013; Henderson et al., 2018). Humans and large animals have relatively thick cortices compared to rodents. The human cerebral cortex is ∼2.5 mm while those of a pig is ∼2.1 mm as opposed to 0.8–1.5 mm in rodents, making the extrapolation of porcine data regarding alcohol induced cortical alterations to humans more representative (Mayhew et al., 1996; Braitenberg and Schüz, 1998; Fischl and Dale, 2000; Vetreno et al., 2017). The porcine brain, with a comparable WM composition and cortical thickness, would provide the means to better understand how alcohol affects the gross and regional morphology of the human brain.

#### Prefrontal Cortex and Corticolimbic System

The prefrontal cortex and corticolimbic system are important because they are heavily implicated in addictive behavior such as AUD as previously discussed. The human brain has a well-defined and studied prefrontal cortex, while there is a significant dispute in the existence of the prefrontal cortex in the rodent brain (Preuss, 1995; Laubach et al., 2018). The region referred to as the “prefrontal cortex” in rodents includes areas such as the anterior cingulate, prelimbic, and infralimbic cortex, which are separate structures from the human prefrontal cortex (Preuss, 1995; Laubach et al., 2018). These inconsistencies in anatomical boundaries generate confusion as unique structural specific activities and responses identified in rodents are then ascribed to the incorrect human brain region (Laubach et al., 2018). Rodents lack the homologous structures to the dorsolateral prefrontal regions, which exhibit pronounced vulnerability to alcohol (Preuss, 1995; Miguel-Hidalgo et al., 2002; Chanraud et al., 2007; Makris et al., 2008; Baranger et al., 2019). Consequently, rodents provide less information pertaining to alcohol induced pathophysiology and overall effect on the dorsolateral prefrontal region (Preuss, 1995). Pig brains, however, have a dorsolateral prefrontal cortex with a comparable prefrontal volume to humans, 10 vs. 12.5% of total brain volume, respectively (Figure 2B; McBride et al., 1999; Jelsing et al., 2006; Kinder et al., 2019).

The pig hippocampus and amygdala corticolimbic regions also resemble those of humans (Figures 2A,C). The pig and human hippocampus are comparable in terms of structure, orientation, and encephalization (Holm and West, 1994; Kinder et al., 2019). It is hidden ventrally within the temporal lobe while the rodent hippocampus lies largely in the dorsal diencephalon rostrally. The orientation and encephalization of the pig hippocampus approximate those of humans, thus allowing a closer simulation of the hippocampus injury that are observed in humans after alcohol exposure. In addition, the volume of individual hippocampal subdivisions and the number of neurons therein are similar between human and pig brains (Holm and West, 1994). Concerning the amygdala, the existence and distribution of somatostatin immunoreactive cell bodies in the pig amygdala is similar to that of humans (Cooper et al., 1981; Rowniak et al., 2008). However, the distribution of somatostatin positive cell bodies is unique in the rat amygdala, with almost no somatostatin expression in the areas that are enriched in the pig and human amygdala. Somatostatin is an inhibitory neuropeptide that regulates the stress response and mood, which are important in the development of AUD (Lin and Sibille, 2013). In addition, alcohol is known to alter the expression of somatostatin receptors and immunoreactivity. This data therefore suggests that the pig might be more predictive in studying the possible role of somatostatin in the human AUD brain (Barrios et al., 2005).

#### Mesolimbic System

Pigs have been used extensively to model the human mesolimbic system due to the high resemblance of pig brains to human brains (Figure 2B). The distribution of 5-HT neurons and the binding patterns of receptors in the medulla of piglets are very similar to those of human infants (Niblock et al., 2005). A receptor binding analysis with a 5-HT agonist revealed that the binding levels for humans and pigs are numerically comparable. Mammals have diverse serotonin receptors in their brain, and although it has not been fully characterized in the pig brain, most subtypes of 5-HT receptors have been identified and consequently cloned (Lind et al., 2007). There is significant evidence that suggests higher homology in the expression of 5-HT receptors and their function in the pig and human brains (Niblock et al., 2005). For example, the human 5HT1B receptor shows higher homology with the pig counterpart at 95% than the 93% homology with mice (Bhalla et al., 2001). A 2% difference in homology can result in markedly differing pharmacological specificity of the receptor. The porcine and human 5HT1B receptors share similar ligand binding properties with affinity for the same agonist and antagonist, while the murine receptor display a prominent difference in binding affinity relative to humans (Bhalla et al., 2001). Similar observations of higher homology in sequence or function are seen in porcine 5HT2 and 5HT6 receptors (Pazos et al., 1984; Johnson et al., 1995). Furthermore, studies of serotonin receptor mRNA expression revealed that pigs have expression of 5HT4 receptors that are present in humans, but absent in the rat (Ullmer et al., 1995; Hirst et al., 2000). In addition, the distribution of dopaminergic dendrites in the pig brain are also more comparable to those in humans. Staining of tyrosine hydroxylase, an enzyme required for DA synthesis, in the porcine brain delineated prominent bundles of dopaminergic neurons that extend from the substantia nigra pars compacta to the pars reticulata in an expression pattern that is more comparable to humans than rats (Ostergaard et al., 1992). The differential distribution of dopaminergic neurons in the rat brain would make it more challenging to understand the dopaminergic circuitry involved with addiction and craving phenotypes in human AUD. This data suggests that the pig mesolimbic system displays a higher level of homology to that of humans in both structure and function.

### Similar Anatomy and Function of the Porcine and Human Liver Results in Comparable Alcohol Metabolism

The anatomy and vasculature of the pig liver is very similar to that of the human liver. This is critical since the liver is the primary site of alcohol elimination (removing > 90% of alcohol) and is significantly affected by alcohol exposure. Alcohol can cause liver cell injury, inflammation, and oxidative stress leading to common liver diseases like cirrhosis, steatosis, and hepatitis (Paton, 2005; Louvet and Mathurin, 2015). Humans and pigs possess a different number of lobes (humans having 4 and pigs having 5), however both human and pig livers are divided into 8 similar segments with comparable vasculature patterns (Court et al., 2003; Nykonenko et al., 2017). Having a similar vascular patterning in the liver between humans and pigs is critical in modeling accurate metabolism and excretion of alcohol. The blood supplied to the liver comes directly from the stomach and small intestine by the portal vein and is dispersed to individual liver segments. There is no significant difference in the blood supply between pigs and humans as the porcine intraparenchymal hepatic arteries follow the portal veins and supply in the same segments, similar to humans. Gross and histological analysis have shown that hepatic portal vein length, wall thickness, and diameter in mature pigs are not significantly different from those of humans (Court et al., 2003; Zhang et al., 2005; Wang et al., 2009). Although the venous drainage in the pig liver is carried out by 4 main hepatic veins as opposed to 3 veins in the human liver, the drainage is released utilizing a similar principle with the separation of hepatic veins according to the segments of the liver, suggesting similarities in outflow of liver blood (Skandalakis et al., 2004; Nykonenko et al., 2017). Humans and pigs both have septal branches that serve as the central axis of blood supply to the acinus (Rappaport, 1976). The acinus is the smallest functional unit of the liver with microcirculation and is extremely relevant to hepatic function as it is oriented around the afferent vascular system (Colorado State University, 2020). On the contrary, the rat liver does not have septal branches of the portal veins or portal veins on the surface of the liver, which are present in both human and pig livers (Figure 1; Bhunchet and Wake, 1998; Teutsch et al., 1999).

Alcohol dehydrogenase and CYP2E1 are mostly expressed in the hepatocytes, so most of the direct cellular toxicity of alcohol is sustained in the liver (Louvet and Mathurin, 2015). Excessive alcohol consumption is the leading cause of liver related death in Western countries. Similar patterns of alcohol induced pathology observed in the brain are also observed in the liver. Alcohol consumption increases ROS and decreases cellular antioxidant levels in the liver, inducing oxidative stress and liver injury (Dey and Cederbaum, 2006). Proinflammatory cytokines and chemokines are markedly increased in the livers of patients with alcoholic hepatitis, a disease caused by chronic alcohol consumption (McClain et al., 1999). Since alcohol induced damage is mediated by enzymes that carry out alcohol metabolism, having similar enzymes is critical in studying alcoholic liver damage. The physiology of the pig liver is more comparable to the human liver because it produces similar enzymes (e.g., CYP2E1 and its isoforms) with greater homology (Figure 1). All the main metabolic activities in human CYP enzymes are present in the porcine liver microsomes including CYP1A, 2A, 2C, 2D, 2E1, and 3A (Anzenbacher et al., 1998; Monshouwer et al., 1998). Rats possess variation in the composition of principal CYP isoforms relative to humans and uninduced rats (wildtypes rats that are not pretreated with agonists to elicit enzyme activity) lack a direct counterpart to some human CYP enzymes. For example, CYP2B is the most prominent isoform in rats responsible for biotransformation of many drugs, while CYP2B is absent in both human and pig liver microsomes. The similar activity of CYP enzymes in pig and human liver is due to the high homology (75%) of the enzymes (Skaanild, 2006). These results indicate that pigs may represent a more appropriate model for oxidative drug metabolisms, such as alcohol, for humans than rats since their enzymes metabolize the same test substrates as the human enzymes (Skaanild, 2006; Skaanild and Friis, 2007). The physiology and pharmacokinetic activities of pigs are more analogous to humans than the traditionally studied rodent models, thus enabling more accurate extrapolations to human studies.

## The Anatomy and Lifespan of Pigs Enables a Closer Inspection of Adolescent Alcohol Exposure

Adolescence is characterized by critical development in the brain with key organizational and structural changes and heightened neural plasticity, rendering it more sensitive to the effects of alcohol (Crews et al., 2016). Exposure to alcohol during this time could potentially cause long-term changes in the cerebral cortex. This explains the higher incidence of alcohol induced deficits in memory, attention, cognitive processing, and language skills in adolescents (Tapert and Schweinsburg, 2005). Adolescent brains are more vulnerable to alcohol induced brain damage compared to adult brains (Crews et al., 2000). This is especially problematic as alcohol is the most widely used intoxicant among adolescents, with 13% of teens engaging in BD within the past 2 weeks (Jacobus and Tapert, 2013). Furthermore, adolescence is a sensitive period for alcohol exposure because early onset of alcohol usage, before the age of 14, is a risk factor for developing AUD later in life (Donovan and Molina, 2011). Although adolescence is a period of higher neurotoxicity, it is also a period of low sensitivity to alcohol induced sedation and motor impairments (White et al., 2002). The combined effects of low alcohol sedation, high risk decisions, and social reward seeking contribute to heavy drinking in adolescents (Crews et al., 2016). As such, adolescence has become a frequently studied period for alcohol exposure in animal models (Crews et al., 2000; White et al., 2002; Teixeira et al., 2014; Fernandes et al., 2018).

During adolescence, the human brain exhibits a loss in GM density due to synaptic pruning, characterized by elimination of weak connections and an increase in WM density due to myelination, a process that enables more efficient communication between brain regions (Giedd, 2004; Gogtay and Thompson, 2010; Doallo et al., 2014). WM fiber tracts are particularly vulnerable because their development is protracted, continuing through early adulthood, when access to alcohol is increased. Smaller WM volumes were observed in adolescents with AUD, particularly in the prefrontal cortex (Schweinsburg et al., 2001; De Bellis et al., 2005; Lebel et al., 2012). Pigs have a similar WM to GM ratio as humans, and the brain development and myelination processes parallel those of humans. The adolescent period, defined by onset of puberty to termination of physical growth, in rats is 1 month while it is 12–13 months in pigs (Reiland, 1978; Jaworska and MacQueen, 2015). The longer adolescent period and overall lifespan (15–27 years) of pigs allows for improved longitudinal studies to assess the chronic effects of alcohol (Jagdale et al., 2019). This would better represent the drinking patterns of humans because AUD is a chronic relapsing disorder (Gonzales et al., 2014). Taken together, these factors indicate that the porcine model provides findings that are more translatable to humans in adolescent alcohol exposure.

## Conclusion

Alcohol is one of the oldest and most prevalent drugs in the history of humankind and has been studied extensively in animal models since the 1940s. Conventionally, rodent models are the most widely used models for AUD, but these models have major inherent biological limitations. While rodent models are useful in understanding the mechanisms of alcohol induced damage, it is challenging for them to replicate the complex physiological, behavioral, and psychological nature of AUD in humans. In this review, we have highlighted some of the advantages of the pig model in studying the pathology and progression of AUD by examining alcohol-induced damage to the brain and liver. Although numerous observational findings were reported in this review, the underlying pathological mechanisms of alcohol induced changes in the brain development are still not fully understood. The reasons for the selective alcohol-induced damage to different regions of the brain remain unclear. Another obscure point is whether the alterations in the brain of AUD subjects are the cause or the consequence of alcohol abuse. The contrasting results and the lack of mechanistic studies of alcohol induced damage are insufficient to conclude which came first. However, the previously described similarities between humans and pigs, suggest that a porcine model will provide invaluable insight into the complex nature of AUD considering the similar rate of alcohol elimination, drinking behavior, tolerance, dependence, and withdrawal symptoms as compared to humans. The porcine model constitutes a new avenue for a more relevant AUD model that can bridge the gap between rodent and human alcohol research.

## Author Contributions

SS was the first author, who conducted literature search, and wrote the entire manuscript. EK generated the figures, conducted critical review, and editing of the manuscript. FW was the mentor of the authors, who played an active role in selecting the topic, writing, editing, and generating the figures. All authors contributed to the article and approved the submitted version.

## Conflict of Interest

The authors declare that the research was conducted in the absence of any commercial or financial relationships that could be construed as a potential conflict of interest.
